# The Extracts Derived from *Artemisia japonica* Thunb. Leaves Mitigate Oxidative Stress and Inflammatory Response Induced by LPS in RAW264.7 Cells through Modulation of the Nrf2/HO-1 Signaling Pathway

**DOI:** 10.3390/molecules29061375

**Published:** 2024-03-20

**Authors:** Yueyu Ye, Xiaomei Li, Man Chen, Xia Wang, Meiya Li, Fusheng Jiang, Xiaobo Zhang, Chunchun Zhang, Shiqing Li

**Affiliations:** 1 College of Pharmaceutical Sciences, Zhejiang Chinese Medical University, Hangzhou 310053, China; yyy13626675378@163.com (Y.Y.); 13858068144@163.com (X.L.); 18386325436@163.com (M.C.); wx66312023@163.com (X.W.); 2 Academy of Chinese Medical Sciences, Zhejiang Chinese Medical University, Hangzhou 310053, China; 20141025@zcmu.edu.cn; 3 College of Life Sciences, Zhejiang Chinese Medical University, Hangzhou 310053, China; jfs1020@163.com

**Keywords:** *Artemisia japonica*, chemical composition, antioxidant, anti-inflammatory, Nrf2/HO-1 signal pathway

## Abstract

*Artemisia japonica* Thunb. has been used as a traditional Chinese medicine and a vegetable for thousands of years in China. However, there are few reports on the chemical composition and biological activity of its leaves. Thus, this study aimed to evaluate the chemical composition, antioxidant and anti-inflammatory effects of water extracts of *A. japonica* leaves and their underlying mechanisms. A total of 48 compounds were identified in the water extract using UPLC-QTOF-MS^2^ analysis, with phenolic acids, particularly chlorogenic acid compounds, being the predominant components. The ethyl acetate fraction (EAF) contained most of the total phenolic content (385.4217 mg GAE/g) and displayed superior antioxidant capacity with the IC_50DPPH•_, IC_50ABTS•+_, and OD_0.5reducing power_ at 10.987 μg/mL, 43.630 μg/mL and 26.883 μg/mL, respectively. Furthermore, EAF demonstrated potent antioxidant and anti-inflammatory effects in LPS-induced RAW264.7 cells by upregulating the Nrf2/HO-1 signal pathway. These findings highlight that *A. japonica* leaves possess remarkable abilities to mitigate inflammation and oxidative stress, suggesting their potential utilization as medicinal agents and food additives for promoting human health.

## 1. Introduction

*Artemisia* L. is a large genus comprising over 500 species, which are mainly found in northern temperate regions, such as North America, Europe, and Asia [1]. The *Artemisia* species plays an important role in the traditional herbal medicine of various countries and is frequently utilized for the treatment of hepatitis, malaria, cancer, infections, and inflammation diseases [1]. Most *Artemisia* plants are rich in essential oil and are used as aromatic plants, which have high application value in the cosmetics and pharmaceutical industry [1,2]. Besides the high-value essential oils, *Artemisia* plants (especially leaves) are also rich in hydrophilic phenolic acids and flavonoids, and the high content of total phenolic compounds in the aqueous extracts of *Artemisia* plants contributes to their high antioxidant activities [3]. Numerous studies have provided evidence of beneficial health effects of consumption of these phenolic compounds due to their antioxidant, anti-inflammation, and vasodilation activities [4], which makes *Artemisia* plants potentially functional foods. Indeed, in addition to being widely used as folk herbal medicine in the treatment of various diseases, many *Artemisia* species are also used as food, spices, condiments, and beverages, on which Trendafilova A. et al. have made a systematic review [5].

*Artemisia japonica* Thunb. (*A. japonica*) is a perennial herb of the *Artemisia* genus, mainly distributed in Asia. According to the Xian Dai Ben Cao Gang Mu, the whole herb of *A. japonica* can be used as medicine, which has the effects of clearing away heat, relieving exterior, cooling blood, and killing insects, and is mainly used for treating cold, fever, strain, cough, hot flashes, heatstroke, malaria, hypertension, aphthous, scabies, and eczema in China [6]. The chemical composition of essential oil from the leaves of *A. japonica* has been extensively studied [7,8]. A series of terpenoids, phenolic acids, and flavonoids were also identified by column chromatography, but most of which were hydrophobic components and lacked pharmacological evaluation [9,10]. On the contrary, several pharmacological studies of *A. japonica* showed that its aqueous extract had great anti-inflammatory, anti-oxidation, and hemostatic activities [11,12]. Genotoxicity and maximum tolerance tests in mice also showed that the water extract of *A. japonica* had low toxicity and high safety [11,12]. Moreover, the purified polysaccharide from *A. japonica* could significantly alleviate collagen-induced arthritis in mice [13]. These results, at least in part, reveal the effectiveness of *A. japonica* as a traditional medicine and the safety of *A. japonica* as an edible and food additive in China, Korea, and Japan [5,14]. However, to the best of our knowledge, no systematic study has been reported on the chemical profile of the water extracts of *A. japonica*. Therefore, additional research is needed to uncover the bioactive components in the water extracts of *A. japonica*.

In the present study, *A. japonica* leaves were extracted with water, and the extract was successively partitioned with ethyl acetate and n-butanol. The content of total phenolics and their antioxidant, anti-inflammatory, and potential mechanisms were analyzed, and the chemical compositions of water extract were investigated by the UPLC-QTOF-MS^2^ technique. We believe that the results from this study will provide sufficient evidence for the further study and utilization of *A. japonica*.

## 2. Results and Discussion

### 2.1. Chemical Composition

**Figure 1:**
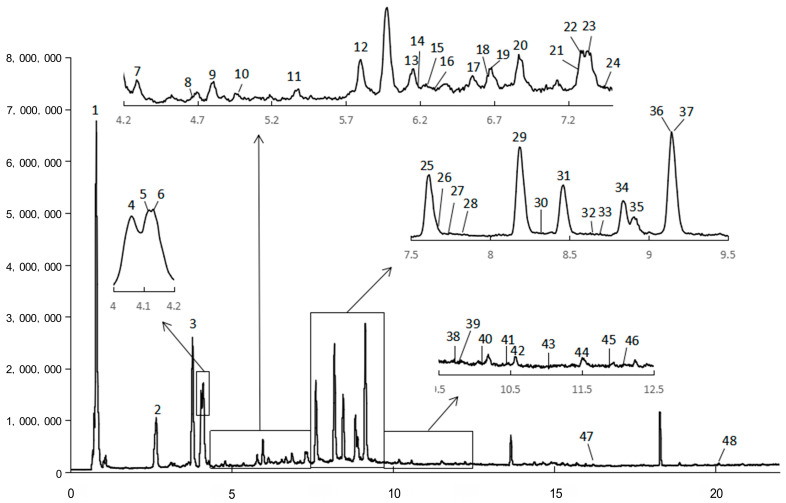
MS chromatography of water extract (WE) of the leaves of *A. japonica* recorded by UPLC-QTOF-MS^2^.

The chemical profiles of *A. japonica* were characterized by UPLC-QTOF-MS^2^ analysis. A total of 48 compounds were detected in the water extract (Table 1, Figure 1). Four compounds were identified by comparing the retention time, high-resolution molecular ions ([M-H]^−^), and major MS^2^ fragment ions of the compounds with those of the standards (Table 1). For example, compound 3 had the same retention time, molecular ion ([M-H]^−^) at *m*/*z* 353, and major fragment at *m*/*z* 191 ([quinic acid-H]^−^ ion) and 179 ([caffeic acid-H]^−^ ion) with the authenticated standard chlorogenic acid, indicating that compound 3 was chlorogenic acid. Similarly, compounds 29, 31, and 36 were positively identified as isochlorogenic acid B, isochlorogenic acid A, and isochlorogenic acid C (Table 1).

The rest of the 44 compounds were tentatively identified according to the retention time, experimental and theoretical molecular ions ([M-H]^−^), major MS^2^ fragment ions, and the MS data in the literature, especially those reported on *Artemisia* species [15,16,17,18,19,20]. For instance, compounds 2, 3, 4, and 9 displayed a [M-H]^−^ ion at *m*/*z* 353, and its MS^2^ spectrum exhibited a base peak at *m*/*z* 191, 191, 179, and 191, respectively. According to the reports, quinic acid was substituted at the 1-OH or 5-OH position and produced a base peak at *m*/*z* 191 [20]. As compound 3 has been authenticated by the standard as 5-*O*-caffeoylquinic acid, therefore, compounds 2 or 9 are likely to be 1-*O*-caffeoylquinic acid. Research indicated that 5-*O*-caffeoylquinic acid has greater hydrophobicity than 1-*O*-caffeoylquinic acid, which ensures that the two isomers can be differentiated on reversed-phase packing [21]; therefore, compound 2 was tentatively characterized as 1-*O*-caffeoylquinic acid. In addition, Zhang Peijie et al. showed that the order of peaks of 3-OH, 4-OH, and 5-OH substitutes of quinic acid on the reversed-phase column was 5-OH, 4-OH, and 3-OH substitutes; meanwhile, the MS^2^ spectrum of 3-*O*-caffeoylquinic acid only gave a [quinic acid–H]^−^ ion at m/z 191 [18]. Therefore, compounds 4 and 9 were tentatively characterized as 4-*O*-caffeoylquinic acid and 3-O-caffeoylquinic acid (Table 1). Another example is that compounds 8, 13, and 14 all give a [M-H]^−^ ion at *m*/*z* 367, with typical MS^2^ fragments at *m*/*z* 193 ([ferulic acid-H]^−^), indicating that the three compounds were feruloylquinic acid isomers. Similar to caffeic acid modification, the elution order of 3-OH, 4-OH, and 5-OH modification of quinic acid by ferulic acid on the reversed-phase column was 5-OH, 4-OH, and 3-OH substitutes [18,20]. Therefore, compounds 8, 13, and 14 were tentatively characterized as 5-*O*-feruloylquinic acid, 4-*O*-feruloylquinic acid, and 3-*O*-feruloylquinic acid. Moreover, compounds 28, 29, 31, 36, and 42 exhibited a [M-H]^−^ ion at *m*/*z* 515 and the major fragments at 353 (loss of caffeoyl), 191 ([M-H]^−^ of quinic acid), and 179 ([M-H]^−^ of caffeic acid), indicating that they were dicaffeoylquinic acid isomers. Compounds 29, 31, and 36 were further identified with the authentic standards as 3,4-*O*-dicaffeoylquinic acid, 3,5-*O*-dicaffeoylquinic acid, and 4,5-*O*-dicaffeoylquinic acid, respectively. The elution order on the reversed-phase column of these three compounds was in accordance with the order reported by Clifford et al. [21]. Additionally, compounds 40, 41, and 43 manifested [M-H]^−^ ion at *m*/*z* 529 and displayed characteristic fragments either at *m*/*z* 367 ([feruloylquinic acid-H]^−^), 335 ([caffeoylquinic acid-H_2_O-H]^−^), 179 ([caffeic acid-H]^−^), or 173 ([quinic acid-H_2_O-H]^−^), indicating that they were caffeoylferuloylquinic acid isomers. Consequently, a total of 48 compounds, including 21 phenolic acids, 18 flavonoids, 3 terpinoids, 2 phenolics, 2 phenylpropanoids, and 2 organic acids, were tentatively identified from the water extract of *A. japonica*. Due to the lack of research on the water-soluble components of *A. japonica*, only three of the above-identified compounds (19, 33, and 48) have been reported in *A. japonica*. However, except for the compound vitexnegheteroin M (39), all other compounds have been reported in other *Artemisia* plants [15,16,17,18,19,20].

### 2.2. Fraction Characterization

**Figure 2:**
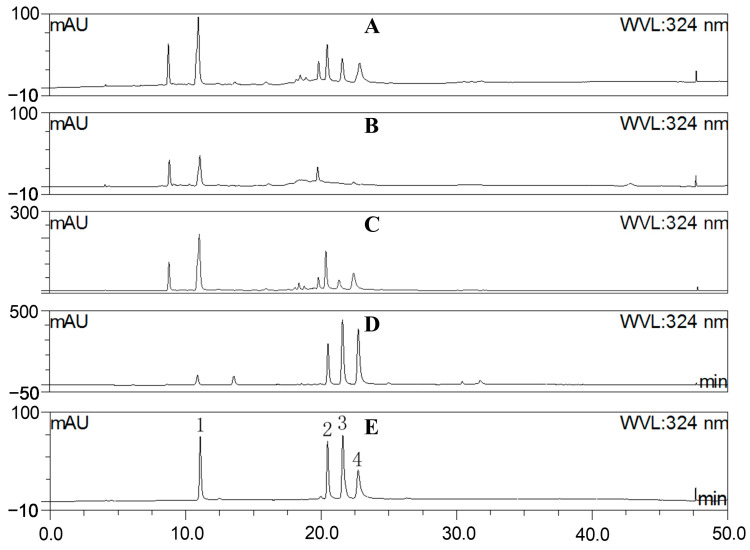
HPLC characterization of (**A**) the water extract of *A. japonica* (WE); (**B**) the water fraction of WE (WE-WF)*;* (**C**) the n-butanol fraction of WE (WE-BF); (**D**) the ethyl acetate fraction of WE (WE-EAF); and (**E**) standards of chlorogenic acids (1. chlorogenic acid; 2. isochlorogenic acid B; 3. isochlorogenic acid A; and 4. isochlorogenic acid C).

The MS chromatography results depicted in Figure 1 and Table 1 indicate that *A. japonica* is rich in phenolic acids and flavonoids, especially chlorogenic acids, which is confirmed by comparing with the standards of chlorogenic acids using the HPLC method (Figure 2). In order to better reveal the anti-inflammatory and antioxidant effects of these components on *A. japonica*, sequential partitioning of the water extract of *A. japonica* leaves was performed using ethyl acetate and n-butanol. As shown in Table 2, most of the components in WE were distributed in water fraction (WF), followed by the n-butanol fraction (BF). The fractions were further characterized by HPLC, and the content of chlorogenic acids was determined. As illustrated in Figure 2 and Table 2, isochlorogenic acids in WE subfractions were predominantly distributed in EAF, while CA was concentrated within BF. The different distribution of these components may contribute to varying pharmacological activities exhibited by different fractions.

### 2.3. Total Phenolics

**Figure 3:**
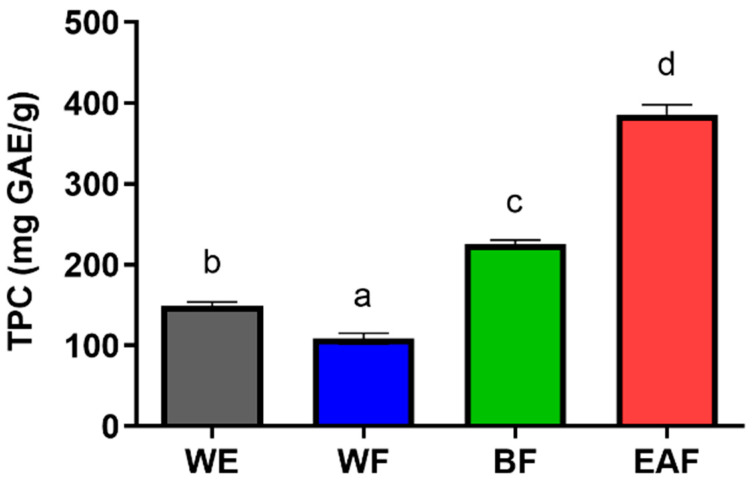
Quantification of total phenolic content in extracts obtained from leaves of *A. japonica*. WE: the water extract of *A. japonica*; WF: the water fraction of WE*;* BF: the n-butanol fraction of WE; EAF: the ethyl acetate fraction of WE. Columns marked with different letters are significantly different from each other (*p* < 0.05).

The total phenolic content (TPC) in crude extracts and its fractions is presented in Figure 3. The TPC in WE reached 149.442 mg GAE/g, while the TPC for different fractions followed the following order: EAF > BF > WE > WF. Notably, the EAF exhibited the highest TPC value of 385.4217 mg GAE/g. These findings highlight *A. japonica* leaves as a promising source of polyphenols, with ethyl acetate serving as an effective solvent for their enrichment.

### 2.4. Comparison of Antioxidant Activity

**Figure 4:**
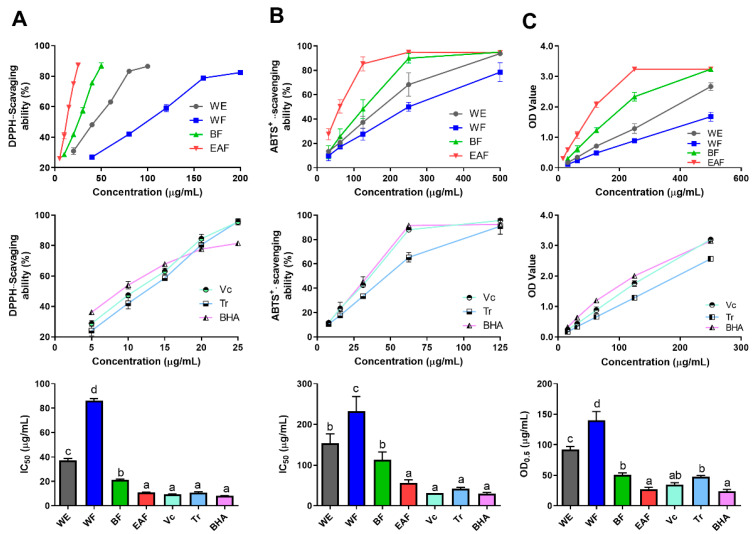
Free radical scavenging ability of the crude extract and fractions of *A. japonica* by the (**A**) DPPH^•^, (**B**) ABTS^+•^, and (**C**) Reducing power assay. WE: the water extract of *A. japonica*; WF: the water fraction of WE*;* BF: the n-butanol fraction of WE; EAF: the ethyl acetate fraction of WE; Vc: vitamin C; Tr: trolox; BHA: butylated hydroxyanisole. The data are presented as the mean ± SD; *n* = 3. Columns marked with different letters are significantly different from each other (*p* < 0.05).

Radical scavenging ability assays (DPPH^•^ and ABTS^+•^) and reducing power assays were used to evaluate the antioxidant activities of the crude extract and fractions of *A. japonica*. For better evaluating the antioxidant activity of the crude extract and fractions of *A. japonica*, Vitamin C (Vc), Trolox (Tr), and Butylated hydroxyanisole (BHA), which are commonly utilized as positive controls for assessing the antioxidant activity of drugs, were all employed as positive controls. The results are presented in Figure 4. It was observed that the scavenging activity of free radicals and reducing the power of the three positive controls showed significant activity, and the overall activity trend followed the following descending order: BHA > Vc > Tr, among which the reducing power of Tr was significantly different from the other two (*p* < 0.05).

As depicted in Figure 4, all fractions showed potential antioxidant activity in a dose-dependent manner, and the antioxidant activity showed a downward trend of EAF > BF > WE > WF in general. In particular, EAF exhibited the strongest free radical scavenging ability and reducing power activity, which were equivalent to those of positive control (Vc and BHA), and with the IC_50 DPPH•_, IC_50 ABTS•+_, and OD_0.5 reducing power_ were at 10.987 μg/mL, 43.630 μg/mL, and 26.883 μg/mL, respectively, while WF gave the lowest antioxidant activity. The results above imply that hot water extraction is a potential method to obtain antioxidant components of *A. japonica*, and the active ingredients are more easily enriched in ethyl acetate and n-butanol fractions.

### 2.5. Anti-Inflammatory Effects on Lipopolysaccharide (LPS)-Induced RAW264.7 Cells

**Figure 5:**
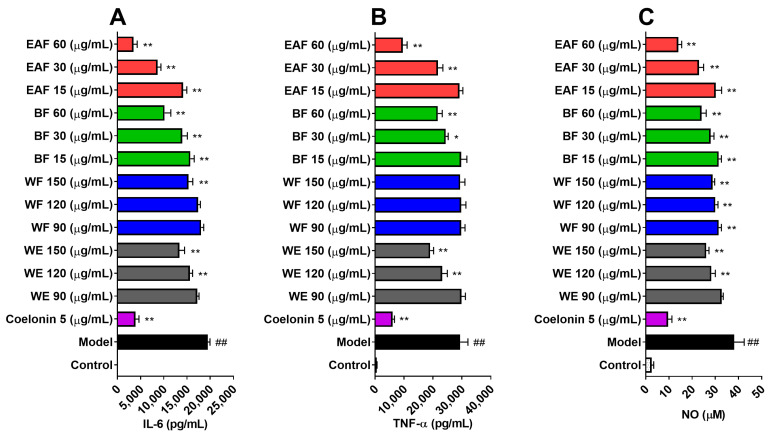
The inhibitory effect of *A. japonica* extracts on LPS-induced inflammatory cytokines and nitric oxide (NO) in RAW264.7 cells. The levels of (**A**) IL-6, (**B**) TNF-α, and (**C**) NO in the supernatant of RAW264.7 cells treated with 100 ng/mL LPS for 24 h were measured after pretreatment with various concentrations of crude extract and fractions of *A. japonica*. The data are presented as the mean ± SD; *n* = 3. ^##^, *p* < 0.01 compare with control; *, *p* < 0.05, **, *p* < 0.01, compare with Model.

LPS stimulation can induce RAW264.7 cells to express and release a variety of proinflammatory cytokines [22]. The levels of interleukin 6 (IL-6) and tumor necrosis factor-ɑ (TNF-ɑ) in the supernatant of RAW264.7 cells were determined after LPS exposure. As shown in Figure 5A,B, the basal levels of IL-6 and TNF-α in RAW264.7 cells were 18.987 pg/mL and 573.315 pg/mL, respectively. Stimulation with 100 ng/mL of LPS for 24 h led to a 1025.7-fold increase in IL-6 and a 51.3-fold increase in TNF-α. Pretreatment with coelonin at 5 μg/mL, previously reported as an effective anti-inflammatory compound [23], dramatically downregulated the expressions of IL-6 and TNF-α. Except for WF, pretreatment with WE and other fractions of *A. japonica* resulted in a concentration-dependent reduction in IL-6 and TNF-ɑ levels. EAF exhibited significant anti-inflammatory activity, leading to an 82.045% decrease in IL-6 and a 67.38% decrease in TNF-ɑ when treated with 60 μg/mL EAF.

### 2.6. Effects on NO and Reactive Oxygen Species (ROS) Production on LPS-Induced RAW264.7 Cells

**Figure 6:**
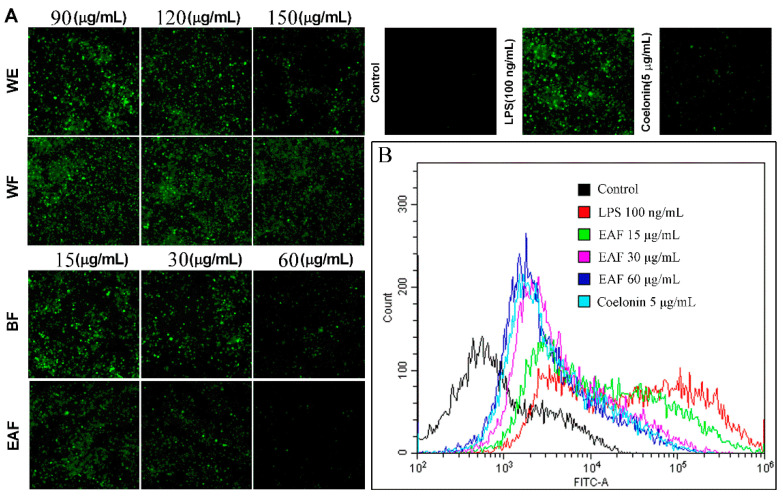
The extracts of *A. japonica* exhibited a dose-dependent inhibition on the levels of ROS in RAW264.7 cells induced by LPS. RAW264.7 cells were pretreated with or without different concentrations of crude extract and fractions of *A. japonica* for 1 h, and following stimulated with 100 ng/mL of LPS for 24 h. After being stained with 10 μM DCFH-DA for 30 min, cells were (**A**) photographed with ImageXpress Micro XLS system and (**B**) determined by flow cytometry.

NO is a multi-effector molecule, which is closely related to inflammation and oxidative stress [24]. LPS can induce over NO and superoxide anion (O_2_^−^) production on RAW264.7 cells through upregulation of inducible nitric oxide synthase (iNOS) and NADPH oxidase, respectively, and these two can form peroxynitrite (ONOO^−^), which mediates the cytotoxic effect of NO [25]. These excessive ROS will damage DNA, lipids, protein, and mitochondria, leading to cell damage and even death. Therefore, inhibition of these ROS can protect cells from oxidative stress. Consistent with previous reports [25], stimulation with 100 ng/mL LPS significantly augmented the levels of NO in the supernatant (from 2.574 μM to 38.130 μM, as shown in Figure 5C), and increased ROS levels within the cells (Figure 6). However, pretreatment with different concentrations of WE and fractions derived from *A. japonica* effectively attenuated NO and ROS levels in a dose-dependent manner. Notably, the NO inhibition and ROS-scavenging active ingredients were mainly enriched in the n-butanol and ethyl acetate fractions. Particularly, the ROS scavenging efficacy of EAF was further evaluated via flow cytometry (Figure 6B), which reconfirmed the remarkable ability of EAF to scavenge ROS.

### 2.7. The Impact of EAF on the Expression of Genes Associated with Inflammation and Oxidative Stress

The binding of LPS to the Toll-like receptor 4 (TLR4) receptor on macrophage surfaces can activate the NF-κB pathway, leading to upregulation of gene expressions such as IL-6, TNF-ɑ, and iNOS, thereby inducing an inflammatory reaction in the body [26]. Simultaneously, LPS can also induce ROS outbreak and oxidative stress in cells by activating the NOX2/ROS pathway [27], interfering with mitochondria [28], and downregulating antioxidant enzymes [29]. An appropriate inflammatory reaction and oxidative stress are beneficial for pathogen elimination, while excessive or chronic inflammation and oxidative stress may contribute to diseases like fibrosis [30], diabetes [31], cancer [32], and Alzheimer’s disease [33]. Those compounds possess antioxidant and anti-inflammatory activities and hold potential value in drug development, as well as functional food additives that promote human health. Evidently, EAF exhibits significant inhibitory activity against LPS-induced secretion of inflammatory factors and the production of free radicals in RAW264.7 cells. This highlights its key role as an anti-inflammatory and antioxidant agent derived from *A. japonica,* with promising prospects for further development and application. Therefore, exploring its molecular mechanism is highly significant.

**Figure 7:**
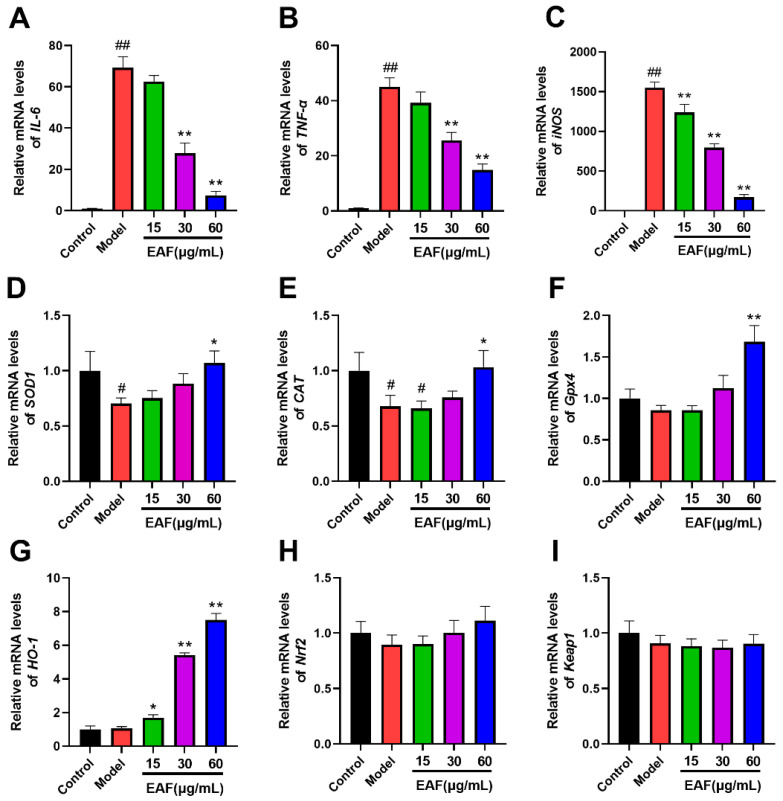
The impact of EAF on the expression of genes related to inflammation and antioxidant activity was assessed using qPCR. Following a 1 h treatment with either vehicle or varying concentrations of EAF, the cells were exposed to LPS at a concentration of 100 ng/mL for 6 h. Subsequently, total RNA was extracted and subjected to qPCR analysis, with expression data normalized to the reference gene β-actin. (**A**–**I**) represents the relative expression levels of *IL-6*, *TNF-ɑ*, *iNOS*, *SOD1*, *CAT*, *Gpx4*, *HO-1*, *Nrf2* and *Keap1* genes, respectively. Data represent the mean ± SD; *n* = 3. ^#^, *p* < 0.05, ^##^, *p* < 0.01 compare with control; *, *p* < 0.05, **, *p* < 0.01, compare with Model.

As shown in Figure 7A,B, the mRNA levels of IL-6 and TNF-ɑ were significantly upregulated by LPS stimulation. However, this upregulation was dose-dependently inhibited by EAF pretreatment, which is consistent with the observed changes in protein levels (Figure 5). These findings provide further evidence for the anti-inflammatory activity of EAF. Moreover, exposure to LPS dramatically increased iNOS mRNA expression (Figure 7C), which is known to be a major source of nitrogen free radicals. Notably, EAF exhibited a strong inhibitory effect on iNOS expression, explaining the corresponding decrease in NO level in the supernatant following EFA treatment (Figure 5). Macrophage oxidative stress is not only caused by excessive production of free radicals but is also associated with an imbalance in the antioxidant system [34]. In line with this concept, superoxide dismutase 1 (SOD1) and catalase (CAT) mRNA levels were significantly decreased upon LPS exposure (Figure 7D,E). Additionally, glutathione peroxidase 4 (Gpx4) mRNA level was also reduced (Figure 7F), all of which could be reversed by 60 μg/mL of EAF pretreatment (Figure 7D,E).

Interestingly, EAF treatment significantly promoted the mRNA level of heme oxygenase-1 (HO-1), with a statistically significant difference observed at 15 μg/mL (Figure 7G). Moreover, the high-dose treatment group at 60 μg/mL exhibited a remarkable increase in HO-1 mRNA level, which was approximately seven-fold higher than that of the LPS model group (Figure 7G). HO-1 is a well-known phase II antioxidant enzyme that can convert hemoglobin into carbon monoxide (CO), ferrous ions (Fe^2+^), and biliverdin, thereby exerting potent antioxidant, anti-inflammatory, and anti-apoptotic effects [35]. Consequently, we speculated that EAF may exhibit its antioxidative activity by upregulating the expression of HO-1.

### 2.8. EAF Exerts Antioxidant and Anti-Inflammatory Effects through the Regulation of the Nrf2/HO-1 Pathway

HO-1 functions as an inducible antioxidant enzyme, which is usually regulated by the nuclear transcription factor Nrf2. Nrf2, a member of the Cap’n’collar (CNC)-BZIP transcription factor family, plays a crucial role in regulating cellular responses to oxidative stress. In normal physiological conditions, Nrf2 is bound to Kelch-like epichlorohydrin-related proteins (Keap1) and remains in a state of low activity. However, under conditions of oxidative stress or other pathological stimuli, modifications occur on the cysteine residues of Keap1, leading to phosphorylation of Nrf2. Consequently, Nrf2 dissociates from the complex and translocates into the nucleus, where it interacts with accessory proteins and recognizes an antioxidant response element (ARE). This interaction activates downstream antioxidant-related genes, including HO-1, SOD, glutamate-cysteine ligase (GCL), and NAD(P)H-quinone oxidoreductase 1 (NQO1). The activation facilitates the removal of reactive oxygen species (ROS) while promoting balance within the body’s antioxidant system [35]. Therefore, we quantified the mRNA levels of Nrf2 and Keap1. However, the results indicated that there were no significant changes in the expression levels of both Nrf2 and Keap1 mRNA following exposure to LPS or EAF treatment (Figure 7H,I).

**Figure 8:**
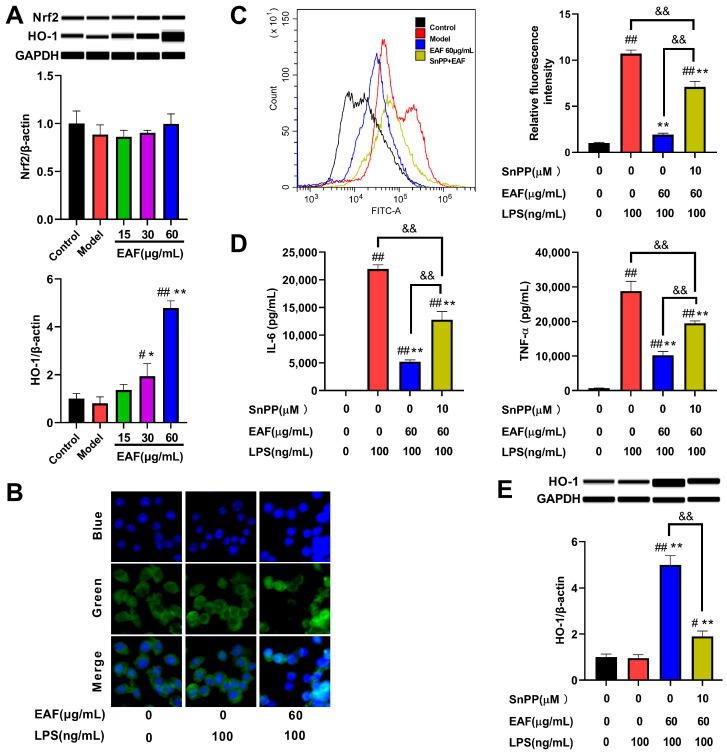
EAF exerts antioxidant and anti-inflammatory effects through the regulation of the Nrf2/HO-1 pathway. The RAW264.7 cells were pretreated with the vehicle, different concentrations of EAF, or the HO-1 inhibitor Snpp (10 μM) combined with EAF (60 μg/mL) for 1 h. Subsequently, they were exposed to 100 ng/mL LPS for either 2 h (to detect Nrf2 nuclear translocation) or 24 h (to assess protein expression, cytokine levels, and ROS levels). (**A**) Protein levels of Nrf2 and HO-1 detected by Simple Western. (**B**) Immunofluorescence analysis of Nrf2 nuclear translocation. (**C**) Flow cytometry analysis of the effect of HO-1 inhibitor intervention on the ROS scavenging activity of EAF. (**D**) Effects of HO-1 inhibitor intervention on the anti-inflammatory activity of EAF. (**E**) The impact of an HO-1 inhibitor on the expression of HO-1 protein induced by EAF. Data represent the mean ± SD, *n* = 3. ^#^, *p* < 0.05, ^##^, *p* < 0.01 compare with control; *, *p* < 0.05, **, *p* < 0.01, compare with Model; ^&&^, *p* < 0.01 compare between two groups.

In order to validate the regulatory effect of EAF on the Nrf2/HO-1 pathway, a Western blot experiment was conducted. The results confirmed that treatment with EAF did not effectively enhance the protein level of Nrf2. However, it dose-dependently promoted the expression of HO-1, particularly in the 60 μg/mL treatment group, which exhibited a 3.454-fold increase compared to the LPS treatment group (Figure 8A). Since Nrf2 is acknowledged as a pivotal regulatory transcription factor for HO-1, immunofluorescence analysis was performed to examine the nuclear translocation of Nrf2. As depicted in Figure 8B, Nrf2 predominantly localized in the cytoplasm in both control and LPS-treated groups; however, after treatment with 60 μg/mL EAF, there was a significant elevation observed in nuclear levels of Nrf2. This suggests that EAF contains components that facilitate the activation of Nrf2.

Recent studies have demonstrated that ROS could activate NF-κB, thereby promoting inflammatory responses and exacerbating tissue damage [36]. Consequently, antioxidant medications can ameliorate excessive inflammatory responses while concurrently reducing oxidative stress within the body, thus facilitating tissue repair [37]. Similarly, the Nrf2/HO-1 pathway not only eliminates ROS but also effectively regulates inflammatory responses [35]. Those compounds that promote HO-1 expression, such as notopterol, nepetoidin B, and ramelteon, can inhibit inflammation, but their effects can be weakened by HO-1 inhibitors [38,39,40]. Therefore, we tested whether EAF exerts antioxidant effects and alleviates inflammation by upregulating the Nrf2/HO-1 pathway. As demonstrated in Figure 8C, exposure to LPS significantly increased intracellular ROS levels. However, pretreatment with 60 μg/mL EAF effectively inhibited this increase in ROS levels, while the scavenging activity of EAF against ROS was found to be attenuated by intervention with an HO-1 inhibitor SnPP. Furthermore, the inhibitory effect of EAF on the secretion of IL-6 and NF-ɑ inflammatory factors induced by LPS was also significantly reduced when co-treated with an HO-1 inhibitor (Figure 8D). These changes in the efficacy of EAF were consistent with alterations observed in the protein level of HO-1 after using SnPP (Figure 8E). Collectively, these findings confirm that EAF alleviates oxidative stress and inflammatory response induced by LPS at least partially through modulation of the Nrf2/HO-1 signaling pathway.

**Figure 9:**
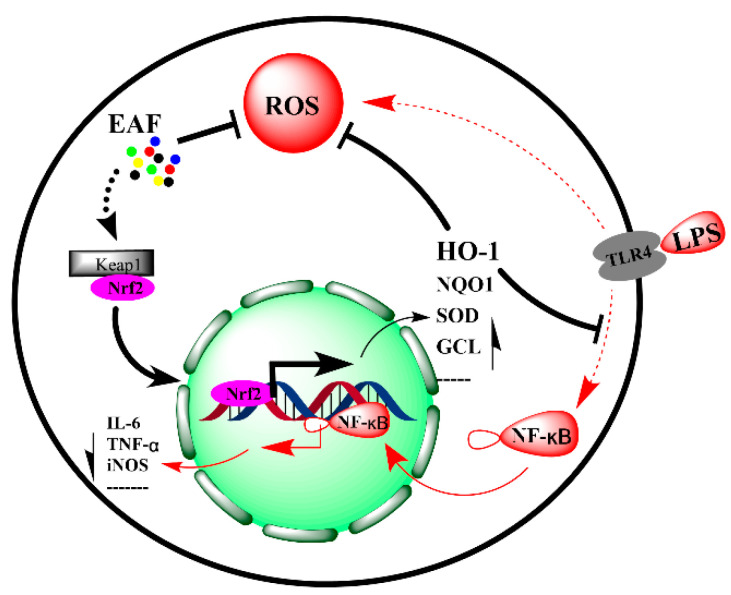
Molecular mechanism of EAF alleviating LPS-induced oxidative stress and inflammatory response in RAW264.7 cells by upregulating Nrf2/HO-1 pathway.

The above results showed that the antioxidant and anti-inflammatory components of *A. japonica* were mainly distributed in the EAF fraction, which is rich in chlorogenic acids, and the total contents of chlorogenic acid (CA), isochlorogenic acid A (IAA), isochlorogenic acid B (IAB), and isochlorogenic acid C (IAC) reached 519.667 mg/g. Numerous publications have shown that AC [41] and IAA [42] have strong antioxidant and cell protective effects. Chlorogenic acid, as the most abundant and effective dietary phenolic compound, not only exhibits remarkable antioxidant activity but also possesses anti-inflammatory properties [41]. According to the literature, the anti-inflammatory effect of chlorogenic acid in various cell inflammation models ranges from 1.77 μg/mL to 708.62 μg/mL [43,44,45]. Therefore, chlorogenic acids are one of the active components of EAF. The current study indicated that besides direct scavenging free radicals, EAF also showed remarkable antioxidant and anti-inflammatory activities in LPS-induced RAW264.7 cells by partially upregulating the Nrf2/HO-1 signal pathway (Figure 9). However, few publications have reported on the ability of these chlorogenic acids to effectively activate Nrf2 nuclear translocation and promote HO-1 expression for alleviating oxidative stress and inflammation in LPS-induced macrophage cells. Therefore, further investigation is warranted to elucidate the constituents contributing to the antioxidant and anti-inflammatory effects of EAF.

## 3. Materials and Methods

### 3.1. Chemicals and Reagents

Chlorogenic acid (CA), butylated hydroxyanisole (BHA), and 2,2-azinobis(3-ethylbenzoth-iazoline-6-sulfonic acid ammonium salt) (ABTS) were purchased from Bide pharmatech Ltd. (Shanghai, China), isochlorogenic acid A (IAA), isochlorogenic acid B (IAB), and isochlorogenic acid C (IAC) were purchased from Chengdu Push Biotechnology Co., Ltd. (Chengdu, China), Folin–Ciocalteu’s phenol reagent was purchased from Solarbio Science & Technology Co., Ltd. (Beijin, China), gallic acid (GAE), 2,2-diphenyl-1-picrylhydrazyl radical (DPPH), trolox (Tr), vitamin C (Vc), 2′,7′-dichlorofluorescin diacetate (DCFH-DA), and thirty percent hydrogen peroxide (30%-H_2_O_2_) were obtained from Meryer Chemical Technology Co., Ltd. (Shanghai, China). The HPLC-grade acetonitrile and formic acid were purchased from Sigma-Aldrich (St. Louis, MO, USA). Ultrapure water was prepared using a Milli-Q purification system (Millipore Laboratory, Bedford, MA, USA). Other chemicals or solvents were of analytical grade and used without further purification.

### 3.2. Plant Material

The leaves of *A. japonica* were collected from Shouning County, Ningde City (Fujian Province, China) in May 2019 and identified by Professor Zhishan Ding of Zhejiang Chinese Medical University. The fresh leaves were cleaned and dried in a constant-temperature oven at 60 °C, then powdered and sieved through an 80 mesh screen. Finally, the powder was sealed and stored in a drier at −40 °C.

### 3.3. Sample Preparation

Leaves powder (10.0 g) was reflux extracted with 100 mL water for 60 min, filtered, and the residue was re-extracted following the same conditions. The supernatants were combined and concentrated to 10 mL with a vacuum rotary evaporator at 45 °C; then, 15 mL ethanol was added. The mixture was placed at 4 °C overnight, then centrifuged at 12,000 rpm for 10 min, and the supernatant was vacuum-dried at 40 °C to obtain the water extract of *A. japonica* (WE). A total of 2.0 g WE was suspended in 40 mL distilled water and partitioned sequentially with ethyl acetate (3 × 40 mL) and n-butanol (3 × 40 mL), respectively. The three fractions were then evaporated to dry under reduced pressure to give ethyl acetate fraction (EAF), n-butanol fraction (BF), and water fraction (WF). These fractions were stored at −40 °C.

### 3.4. HPLC Characterization

All extracts were prepared with 50% acetonitrile at a concentration of 1 mg/mL and analyzed by HPLC using the UltiMate 3000 high-performance liquid chromatography system (Dionex Corp., Sunnyvale, CA, USA). Method S1 (Supplementary Materials) contains specific chromatographic conditions.

### 3.5. UPLC-QTOF-MS^2^ Conditions

The samples were subjected to the UPLC-QTOF-MS^2^ system, which consisted of an ACQUITY ultraperformance liquid chromatography instrument (Waters Corporation, Milford, MA, USA) and a SYNAPT G2-Si Q-TOF Mass Spectrometer (Waters Corporation, Manchester, UK), to reveal the chemical composition of *A. japonica* leaves. The chromatographic separation conditions and mass spectrometry analysis methods are detailed in Method S2 (Supplementary Materials).

### 3.6. Determination of Total Phenolic Content (TPC)

The TPC in fractions was determined by the Folin–Ciocalteu’s method as reported by [15] with gallic acid as standard (*Y* = 9.3253*X* + 0.0052, R^2^ = 0.9998). The TPC was expressed as a milligram of gallic acid equivalent per gram of fraction (mg GAE/g). The whole sample was tested in triplicate.

### 3.7. Radical Scanvenging Ability Assays

#### 3.7.1. DPPH^•^ Scanvenging Ability

The DPPH^•^ scavenging activity was determined following the method of [15]. Briefly, 100 μL of the sample was mixed with an equal volume of freshly prepared DPPH^•^ solution (0.1 mM). After incubating in the dark at room temperature for 30 min, the absorbance was recorded at 510 nm. Vc, Te, and BHA were used as positive controls. IC_50_ value was calculated, and the whole sample was tested in triplicate.

#### 3.7.2. Reducing Power

The reducing power activity was determined according to the method reported by [46]. Absorbance was measured at 700 nm, and the OD_0.5_ value (the concentration of sample absorbance of 0.500) was calculated. Vc, Te, and BHA were used as positive controls, and all the tests were made in triplicate.

#### 3.7.3. ABTS^+•^ Scavenging Activity

The ABTS^+•^ scavenging activity was conducted following the reference [15] and with modification. Briefly, 20 μL of the sample was mixed with 180 μL of freshly prepared ABTS^+•^ solution, and the absorbance was measured at 734 nm after 6 min. Using Vc, Te, and BHA as positive controls, the IC_50_ value was calculated.

### 3.8. Anti-Inflammatory and Reactive Oxygen Species (ROS) Scavenging Activities on LPS-Induced RAW264.7 Cells

#### 3.8.1. Nitric Oxide (NO) and Cytokines Level in Supernatants

RAW264.7 cells (American Type Culture Collection) were cultured in DMEM media supplemented with 10% FBS and 1% penicillin-streptomycin. RAW264.7 cells (5 × 10^5^ cells/mL) were seeded in 96-well plates and incubated overnight. The drugs and extracts of *A. japonica* were prepared as high-concentration stock solutions using DMSO, followed by dilution into a series of treatment concentrations with serum-containing DMEM medium to achieve a final DMSO content of 0.1%. The medium containing 0.1% DMSO was utilized as the reagent control, while the coelonin treatment group at a concentration of 5 μg/mL was used as the positive control. Subsequently, the medium in the 96-well plate was discarded and replaced with the aforementioned diluted drugs. After incubation for 1 h, LPS at a final concentration of 100 ng/mL was added to stimulate the cells for 24 h. Then, the culture supernatants were collected for detection of the NO level using the Griess reagent method [47], and the interleukin-6 (IL-6) and tumor necrosis factor-ɑ (TNF-ɑ) levels in the supernatants were analyzed by cytometric beads array (CBA) method [23].

#### 3.8.2. Determination of Intracellular ROS

RAW264.7 cells were seeded in 96-well and 6-well plates and treated as NO assay. After 24 h treatment, cells were stained with 2′,7′-dichlorofluorescin diacetate (DCFH-DA, 10 μM) for 30 min at 37 °C. The medium was then removed and washed with serum-free DMEM three times. Cells in 96-well plates were photographed by ImageXpress Micro XLS system (Molecular Devices LLC, Sunnyvale, CA, USA). Cells in 6-well plates were collected and resuspended in PBS and immediately analyzed on a BD Accuri™ C6 flow cytometer (BD, Ann Arbor, MI, USA).

### 3.9. Real-Time Fluorescence Quantitative PCR (qPCR) Analysis

RAW264.7 cells (5 × 10^5^ cells/mL) were seeded in 6-well plates and pretreated with either vehicle (containing 0.1% DMSO in the medium) or various concentrations of extracts, with or without the HO-1 inhibitor (SnPP) for 1 h. Subsequently, the cells were exposed to 100 ng/mL LPS for 6 h. Following our previously published protocol [23], the cells were collected, and total RNA was extracted with Trizol reagent (BS259A; Biosharp, Hefei, China). The RNA concentration was measured using a trace nucleic acid detector (Thermo Fisher Scientific, Waltham, MA, USA), and cDNA was obtained using a reverse transcription kit (RiboBio, Guangzhou, China). The mRNA expression levels of IL-6, TNF-ɑ, inducible nitric oxide synthase (iNOS), Kelch-1ike ECH-associated protein l (Keap1), nuclear factor erythroid 2-related factor 2 (Nrf2), catalase (CAT), superoxide dismutase 1 (SOD1), glutathione peroxidase 4 (Gpx4), and heme oxygenase (HO-1) were detected through qPCR using specific primers listed in Table S1. The expression levels of each gene were calculated relative to β-actin according to the 2^−ΔΔCT^ method and normalized relative to the control group.

### 3.10. Western Blot Analysis

Treated cells were harvested and lysed with RIPA buffer (78503; Thermo Fisher Scientific) supplemented with a phosphatase inhibitor (41659200; Roche) and a protease inhibitor (18065900; Roche, Mannheim, Germany). Nuclear and cytoplasmic proteins were separated using a nucleoprotein and plasma protein extraction kit (P0027; Beyotime, Beijing, China). The protein levels were subsequently determined using a Simple Western System (ProteinSimple, San Jose, CA, USA). Briefly, the protein concentrations were adjusted with buffer solution, mixed with the loading buffer, denatured, and subjected to capillary electrophoresis. After the blocking step was performed, followed by incubation with primary antibodies, such as anti-GAPDH (A19056; ABclonal, Wuhan, China), anti-Nrf2 (A0674; Abclonal, Wuhan, China), or anti-HO-1 (A1346; ABclonal, Wuhan, China), and then incubated with secondary antibody. Finally, the samples were imaged and quantified using Simple Western System software 6.3.0 (ProteinSimple, San Jose, CA, USA) [23].

### 3.11. Immunofluorescence Analysis

RAW264.7 cells were preincubated with a vehicle solution (containing 0.1% DMSO in the medium) or with EAF (60 μg/mL) for 1 h, followed by stimulation with LPS (100 ng/mL) for 2 h. The cells were fixed using 4% paraformaldehyde and permeabilized with Triton-100 (0.1%). After overnight incubation with the primary antibody against Nrf2, the cells were washed and further incubated with goat anti-rabbit IgG H&L (Alexa Fluor^®^ 488) (ab150073; Abcam, Cambridge, MA, USA) for an additional hour. After DAPI (C1002; Beyotime, Shanghai, China) staining, the cells were imaged with an ImageXpress Micro Confocal High-Content Cell Imaging System (Molecular Devices).

### 3.12. Statistical Analysis

Data were presented as the mean ± standard deviation (SD) in triplicate. Statistical significance was considered at *p* < 0.05, followed by a one-way analysis of variance (ANOVA) with Tukey’s post hoc test. IC_50_ values, statistical analyses, and figures were prepared using GraphPad Prism software (Version 6.0, Graphpad Software Inc., San Diego, CA, USA).

## 4. Conclusions

In summary, the water extract of *A. japonica* is abundant in phenolic compounds that are primarily enriched in the ethyl acetate fraction, which exhibits the most potent free radical scavenging activity in vitro and also possesses significant antioxidant and anti-inflammatory properties in LPS-induced RAW264.7 cells. Notably, the ethyl acetate fraction prominently activates the Nrf2/HO-1 pathway and partially relies on this pathway to alleviate oxidative stress and inflammatory responses induced by LPS. However, the specific active compound responsible for these effects remains unknown. Apparently, these findings provide substantial support for the potential application of *A. japonica* as a medicinal and health food additive to enhance human well-being by mitigating oxidative stress and inflammatory risks.

## Figures and Tables

**Table 1:** Chemical components in the water extract of *A. japonica*.

Peak	Rt (min)	Exptl. [M-H]−	Theor. [M-H]−	Error (ppm)	Fragments	Chemical Formula	Tentative Identification	Family (Subclass)
1	0.8136	191.0558	191.0561	−1.6994	173.04667, 127.03973, 111.04506, 109.02987, 93.03444, 87.00940, 85.02896	C_7_H_12_O_6_	Quinic acid	Organic acids
2	2.6626	353.0890	353.0878	3.3511	191.05583, 179.03461, 135.04426, 127.04007, 85.02972	C_16_H_18_O_9_	1-*O*-Caffeoylquinic acid	Phenolic acid (Cinnamic acid)
3	3.7991	353.0879	353.0878	0.3652	215.05802, 191.05548, 179.03509, 135.04519, 127.03939, 85.02859	C_16_H_18_O_9_	5-*O*-Caffeoylquinic acid ^1^	Phenolic acid (Cinnamic acid)
4	4.0636	353.0879	353.0878	0.1970	191.05580, 179.03444, 173.04576, 135.04410, 127.04087	C_16_H_18_O_9_	4-*O*-Caffeoylquinic acid	Phenolic acid (Cinnamic acid)
5	4.1107	593.1528	593.1512	2.6666	463.09090, 408.03010, 239.05638, 163.03970	C_27_H_30_O_15_	Kaempferol-3-neohesperidoside	Flavonoid (Flavonol)
6	4.1301	177.0558	177.0557	0.4088	163.04033, 149.06059	C_10_H_10_O_3_	4-Hydroxy-3-methoxycinnamaldehyde	Phenolics
7	4.3013	179.0343	179.0350	−3.5685	161.02510, 135.04402, 133.03013, 93.03377	C_9_H_8_O_4_	Caffeic acid	Phenolic acid (Cinnamic acid)
8	4.6835	367.1053	367.1035	4.8973	193.05058, 134.03673	C_17_H_20_O_9_	5-*O*-Feruloylquinic acid	Phenolic acid (Cinnamic acid)
9	4.7968	353.0889	353.0878	2.9779	191.05601	C_16_H_18_O_9_	3-*O*-Caffeoylquinic acid	Phenolic acid (Cinnamic acid)
10	5.0113	337.0945	337.0929	4.6514	191.05673	C_16_H_18_O_8_	p-Coumaroylquinic acid	Phenolic acid (Cinnamic acid)
11	5.3766	593.1537	593.1512	4.2190	515.12411, 353.08952, 191.05683	C_27_H_30_O_15_	Luteolin-7-*O*-rutinoside	Flavonoid (Flavone)
12	5.8298	357.1207	357.1191	4.5729	195.06719, 151.07656	C_16_H_22_O_9_	Sweroside	Terpenoids (Iridoid)
13	6.1530	367.1042	367.1035	1.9545	193.05224, 173.04529, 134.03753	C_17_H_20_O_9_	4-*O*-Feruloylquinic acid	Phenolic acid (Cinnamic acid)
14	6.2482	367.1042	367.1035	2.0352	193.05114, 173.04534, 134.03790	C_17_H_20_O_9_	3-*O*-Feruloylquinic acid	Phenolic acid (Cinnamic acid)
15	6.2988	563.1430	563.1406	4.2293	447.0958	C_26_H_28_O_14_	Isoshaftoside	Flavonoid (Flavone)
16	6.3239	609.1474	609.1461	2.0435	563.14221, 447.09580, 271.02368, 161.02540	C_27_H_30_O_16_	Kaempferol-3-*O*-glucosyl (1-2) galactoside	Flavonoid (Flavonol)
17	6.5276	463.0897	463.0882	3.3226	301.03689	C_21_H_20_O_12_	Quercetin-3-*O*-β-d-glucoside isomer	Flavonoid (Flavonol)
18	6.6653	341.1250	341.1242	2.5118	193.05047, 149.05998, 133.02946	C_16_H_22_O_8_	citrusin D	Phenolics
19	6.6808	193.0507	193.0506	0.4165	149.05998, 134.03643, 133.02946	C_10_H_10_O_4_	Ferulic acid ^2^	Phenolic acid (Cinnamic acid)
20	6.8748	463.0882	463.0882	0.1086	301.03617, 300.02932, 283.02590, 151.00372	C_21_H_20_O_12_	Quercetin-7-*O*-β-d-glucopyranoside	Flavonoid (Flavonol)
21	7.2931	609.1489	609.1461	4.6232	537.12653, 287.05783, 175.02609, 151.00379, 135.04537	C_27_H_30_O_16_	Kaempferol-3-*O*-glucosyl(1-2)galactoside isomer	Flavonoid (Flavonol)
22	7.3235	463.0891	463.0882	2.0099	300.03021, 151.00344	C_21_H_20_O_12_	Quercetin-3-*O*-β-d-glucoside isomer	Flavonoid (Flavonol)
23	7.4854	593.1530	593.1512	3.0739	533.13349	C_27_H_30_O_15_	Luteolin-7-rutinoside isomer	Flavonoid (Flavone)
24	7.5717	463.0893	463.0882	2.3953	301.03636, 255.03164	C_21_H_20_O_12_	Quercetin-3-*O*-β-d-glucoside isomer	Flavonoid (Flavonol)
25	7.6157	461.0730	461.0725	0.9859	285.04095, 201.02004, 151.00407	C_21_H_18_O_12_	Kaempferol-3-*O*-glucuronide	Flavonoid (Flavonol)
26	7.6969	447.0950	447.0933	3.7809	323.07978, 285.04160	C_21_H_20_O_11_	Kaempferol-7-*O*-glucoside	Flavonoid (Flavonol)
27	7.7526	463.1240	463.1246	−1.2866	229.01497	C_22_H_24_O_11_	Eriodictiol-7-glucoside	Flavonoid (Dihydroflavone)
28	7.8486	515.1203	515.1195	1.5494	353.08896, 315.05243, 191.05748, 179.03625	C_25_H_24_O_12_	Dicaffeoylquinic acid isomer	Phenolic acid (Cinnamic acid)
29	8.1879	515.1190	515.1195	−0.8976	353.08833, 335.07794, 191.05580	C_25_H_24_O_12_	3,4-*O*-Dicaffeoylquinic acid ^1^	Phenolic acid (Cinnamic acid)
30	8.3397	487.1262	487.1246	3.2261	295.06312, 191.05590	C_24_H_24_O_11_	Acacetin-7-*O*-(6″-*O*-acetyl)-β-d-glucopyranoside	Flavonoid (Flavone)
31	8.4579	515.1179	515.1195	−3.0203	353.08884, 335.08051, 191.05588, 179.03469, 173.04548, 161.02510, 135.04450	C_25_H_24_O_12_	3,5-*O*-Dicaffeoylquinic acid ^1^	Phenolic acid (Cinnamic acid)
32	8.6790	353.0888	353.0878	2.7676	181.04996	C_16_H_18_O_9_	Scopolin	phenylpropanoid (Coumarin)
33	8.7417	187.0977	187.0976	0.5508	125.09792	C_9_H_16_O_4_	Azelaic acid ^2^	Organic acids
34	8.8887	431.1005	431.0984	4.8819	269.04707, 268.03982	C_21_H_20_O_10_	Apigenin-7-*O*-glucoside	Flavonoid (Flavone)
35	8.9063	269.0463	269.0455	2.7163	227.03775, 151.00411, 117.03409	C_15_H_10_O_5_	Apigenin	Flavonoid (Flavone)
36	9.1441	515.1187	515.1195	−1.5220	353.08859, 335.07898, 191.05625, 179.03476, 173.04525, 161.02518, 135.04435	C_25_H_24_O_12_	4,5-*O*-Dicaffeoylquinic acid ^1^	Phenolic acid (Cinnamic acid)
37	9.1444	161.0244	161.0244	0.0043	137.02529, 93.03450	C_9_H_6_O_3_	7-Hydroxycoumarin	phenylpropanoid (Coumarin)
38	9.7478	461.1080	461.1089	−2.1180	323.07777	C_22_H_22_O_11_	Diosmetin-7-*O*-β-d-glucopyranosid	Flavonoid (Flavone)
39	9.8104	549.1991	549.1978	2.4796	387.16709, 207.10416, 161.02516	C_27_H_34_O_12_	Vitexnegheteroin M	Phenolic acid (Cinnamic acid)
40	10.1438	529.1362	529.1351	2.0388	367.10485, 335.08081, 301.03970, 173.04600	C_26_H_26_O_12_	Caffeoylferuloylquinic acid isomer	Phenolic acid (Cinnamic acid)
41	10.4875	529.1360	529.1351	1.6767	499.12753, 367.10461, 135.04617	C_26_H_26_O_12_	Caffeoylferuloylquinic acid isomer	Phenolic acid (Cinnamic acid)
42	10.5746	515.1200	515.1195	0.9653	353.08906, 191.05667, 179.03605, 173.04642	C_25_H_24_O_12_	Dicaffeoylquinic acid isomer	Phenolic acid (Cinnamic acid)
43	11.0945	529.1361	529.1351	1.7751	367.10666, 335.08190, 271.09856, 179.03642, 173.04594, 135.04597	C_26_H_26_O_12_	Caffeoylferuloylquinic acid isomer	Phenolic acid (Cinnamic acid)
44	11.5266	207.0658	207.0663	−2.1425	179.03595, 135.04408	C_11_H_12_O_4_	Ethyl caffeate	Phenolic acid (Cinnamic acid)
45	11.9334	677.1515	677.1512	0.5120	555.11800, 515.12006, 353.08866, 335.07949, 191.05651, 179.03505, 173.04614	C_34_H_30_O_15_	3,4,5-Tricaffeoylquinic acid	Phenolic acid (Cinnamic acid)
46	12.1084	283.0621	283.0612	3.1644	268.03964	C_16_H_12_O_5_	Acacetin	Flavonoid (Flavone)
47	16.3310	293.1760	293.1758	0.6382	236.10563, 221.15397, 220.14706, 192.11523	C_17_H_26_O_4_	7β-Acetoxy-2β-hydroxyoplopenone	Terpenoids (Sesquiterpenoids)
48	20.1050	233.1552	233.1547	2.1908	221.15524	C_15_H_22_O_2_	Artemisic acid ^2^	Terpenoids (Sesquiterpenoids)

R_t_ represents retention time; Exptl. [M-H]^−^ and Theor. [M-H]^−^ represent experimental and theoretical *m*/*z* of molecular ions, respectively; the formulas were calculated using Masslynx 4.1 software with mass accuracy of less than 5 ppm between experimental and theoretical *m*/*z*. ^1^ Compound was positively identified by standards. ^2^ Compound was already reported in *A. japonica*.

**Table 2:** Extraction yield and chlorogenic acids content in different fractions.

Fractions	Yield (%)	CA (mg/g)	IAA (mg/g)	IAB (mg/g)	IAC (mg/g)
WE	25.400 ± 1.600 *	45.206 ± 2.494	14.880 ± 1.363	18.610 ±2.459	23.048 ± 1.757
WF	72.719 ± 2.719	20.856 ± 3.700	0.000	0.000	1.957 ± 0.099
BF	21.983 ± 0.715	106.814 ± 4.122	19.115 ± 0.545	53.721 ± 1.790	41.916 ± 2.807
EAF	4.417 ± 0.775	28.677 ± 4.714	188.537 ± 1.521	100.851 ± 2.353	201.602 ± 2.866

* represents the percentage of dry weight of leaves; otherwise it is the percentage of content of extracts. CA: chlorogenic acid; IAA: isochlorogenic acid A; IAB: isochlorogenic acid B; IAC: isochlorogenic acid C.

## Data Availability

Data are contained within the article and Supplementary Materials.

## Supplementary Materials

The following supporting information can be downloaded at https://www.mdpi.com/article/10.3390/molecules29061375/s1, Method S1. HPLC characterization. Method S2. UPLC-QTOF-MS^2^ conditions. Table S1: Primer list for qPCR.
