# Supplementary material for: The Extracts Derived from Artemisia japonica Thunb. Leaves Mitigate Oxidative Stress and Inflammatory Response Induced by LPS in RAW264.7 Cells through Modulation of the Nrf2/HO-1 Signaling Pathway
Source: Molecules. 2024 Mar 20;29(6):1375. doi: 10.3390/molecules29061375 (PMC10974559; doi:10.3390/molecules29061375)
Supplement: Supplementary file 1 [file molecules-29-01375-s001.zip › molecules-2908454-supplementary.pdf]

Method S1. HPLC characterization.

HPLC analysis was conducted using an UltiMate 3000 HPLC system (Dionex Corp., Sunnyvale, CA, USA) with an Inertsil® ODS-SP C18 column (4.6 mm×250 mm, 5 μm, Shimadzu Corporation, Tokyo, Japan). The mobile phase consisted of acetonitrile (A) and 0.1% (V/V) formic acid in deionized water (B). A gradient elution program was employed as follows: 0-5 min - 5-15% A; 5-12 min - 15% A; 12-14 min - 15-25% A; 14-24 min -25% A; and finally from minute 24 to minute 44 the concentration of solvent A increased linearly from a starting point of 25 % to reach a final concentration of 95 %. The flow rate was maintained at a constant value of 1 mL/min. The column temperature was set at 30 °C, while detection occurred at 324 nm wavelength. Each sample injection volume amounted to10 μL. All extracts were prepared by diluting the samples with 50 % acetonitrile solution to achieve a final concentration of 1 mg/mL.

Method S2. UPLC-QTOF-MS^2 conditions.

The UPLC-QTOF-MS^2 system comprised an ACQUITY UPLC instrument (Waters Corporation, Milford, MA, USA) and a SYNAPT G2-Si Q-TOF Mass Spectrometer (Waters Corporation, Manchester,UK). The separation was conducted using a Waters ACQUITY Cortecs T3 column (2.1×100 mm, 1.7μm, Waters, UK) with Cortecs T3 Van Guard (2.1×50 mm, 1.7 μm) at 40 °C. The mobile phase consisted of acetonitrile (A) and 0.1% (V/V) formic acid in deionized water (B) was employed. The gradient elution program consisted of the following steps: 0-3 min, 95% B; 3-8.5 min, 95-80% B; 8.5-13 min, 80-70% B; 13-19 min, 70-40% B; 19-23 min, 40 -5 % B; 23-25 min, 5 % B. The flow rate was maintained at a constant value of 0.3 mL/min throughout the analysis. The column temperature was precisely controlled at 40 °C and the injection volume was 2 μL. In electrospray ionization (ESI)-MS analysis, the source was operated in the negative ion mode with a capillary voltage of 3.0 kV. The ionization temperature and desolvation

temperature were set at 120 °C and 500 °C, respectively. Two channels of MS measurements were recorded: the first channel collected MS signals ranging from m/z 50 to 1200, while the second channel provided fragmentation information for compound identification by collecting mass fragments within the same m/z range using a collision energy of 30 eV.

Table S1. Primer list for qPCR.

Genes	Forward primer (5′-3′)	Reverse primer (5′-3′)
IL-6	GAGACTGGGGATGTCTGTAGC	TCACCAGCATCAGTCCCAAG
TNF-α	CGAGTGACAAGCCTGTAGCCC	GGGCAGCCTTGTCCCTTGA
iNOS	CTTGGAGCGAGTTGTGGATTGTC	TAGGTGAGGGCTTGGCTGAGTGA
SOD1	GAGCATTCCATCATTGGCCG	ACTGCGCAATCCCAATCACT
CAT	CACTGACGAGATGGCACACT	TGTGGAGAATCGAACGGCAA
Gpx4	CCTTCCCCTGCAACCAGTTT	GTGGGCATCGTCCCCATTTA
Nrf2	AGCCAGCTGACCTCCTTAGA	AGTGACTGACTGATGGCAGC
Keap1	GTAGGCCGCCTCATCTACAC	TGGAGTCAGTGTTGCCATCC
HO-1	CAGAAGAGGCTAAGACCGCC	TCTGACGAAGTGACGCCATC
Actin	TGGAGCAAACATCCCCCAAA	CGCGACCATCCTCCTCTTAG
